# Anodal transcranial direct current stimulation of parietal cortex enhances action naming in Corticobasal Syndrome

**DOI:** 10.3389/fnagi.2015.00049

**Published:** 2015-04-14

**Authors:** Rosa Manenti, Marta Bianchi, Maura Cosseddu, Michela Brambilla, Cristina Rizzetti, Alessandro Padovani, Barbara Borroni, Maria Cotelli

**Affiliations:** ^1^Neuropsychology Unit, IRCCS Istituto Centro San Giovanni di Dio FatebenefratelliBrescia, Italy; ^2^Centre for Aging Brain and Neurodegenerative Disorders, Neurology unit, University of BresciaBrescia, Italy; ^3^S. Isidoro Hospital, FERB Onlus, Trescore Balneario (Bergamo)Italy

**Keywords:** language disorders, non invasive brain stimulation, parietal cortex, dementia, frontotemporal dementia (FTD), cognitive disorders

## Abstract

**Background**: Corticobasal Syndrome (CBS) is a neurodegenerative disorder that overlaps both clinically and neuropathologically with Frontotemporal dementia (FTD) and is characterized by apraxia, alien limb phenomena, cortical sensory loss, cognitive impairment, behavioral changes and aphasia. It has been recently demonstrated that transcranial direct current stimulation (tDCS) improves naming in healthy subjects and in subjects with language deficits.

**Objective**: The aim of the present study was to explore the extent to which anodal tDCS over the parietal cortex (PARC) could facilitate naming performance in CBS subjects.

**Methods**: Anodal tDCS was applied to the left and right PARC during object and action naming in seventeen patients with a diagnosis of possible CBS. Participants underwent two sessions of anodal tDCS (left and right) and one session of placebo tDCS. Vocal responses were recorded and analyzed for accuracy and vocal Reaction Times (vRTs).

**Results**: A shortening of naming latency for actions was observed only after active anodal stimulation over the left PARC, as compared to placebo and right stimulations. No effects have been reported for accuracy.

**Conclusions**: Our preliminary finding demonstrated that tDCS decreased vocal reaction time during action naming in a sample of patients with CBS. A possible explanation of our results is that anodal tDCS over the left PARC effects the brain network implicated in action observation and representation. Further studies, based on larger patient samples, should be conducted to investigate the usefulness of tDCS as an additional treatment of linguistic deficits in CBS patients.

## Introduction

Frontotemporal dementia (FTD) is an umbrella term for a clinically heterogeneous group of disorders that primarily affects the frontal and temporal lobes of the brain, areas generally associated with personality, behavior and cognitive impairments. (Grossman et al., 1996; Hodges and Patterson, 1996; Neary et al., 1998; Gorno-Tempini et al., 2004). Furthermore, several studies have proved that two extrapyramidal syndromes such as Progressive Supranuclear Palsy (PSP) and Corticobasal Syndrome (CBS) overlap both clinically and neuropathologically with FTD (for a review see Kertesz and Munoz, 2004). In particular, CBS is a clinical entity characterized by a relatively specific pattern of cortical atrophy (McKhann et al., 2001) and basal ganglia dysfunction as reflected by varying combination of stiffness, clumsiness, dystonia, ideomotor apraxia, alien limb phenomenon, cortical sensory loss, visual or sensory hemi neglect, myoclonus and language deficits (Armstrong et al., 2013).

CBS is associated with a pattern of brain atrophy that involves prefrontal and parietal areas, as well as other cortical and subcortical structures involved in action organization and motor control (Borroni et al., 2008; Whitwell et al., 2010; Armstrong et al., 2013). CBS demonstrated several distinct clinical syndromes, leading to describe CBS as linked with a number of diverse pathologies and characterized by high heterogeneity (Ling et al., 2010; Hassan et al., 2011). Language difficulties have been demonstrated in 40% of CBS patients at presentation and in 52% of the patients over disease course (Armstrong et al., 2013). Language disorder in CBS is characterized by speech production failure with apraxia of speech and/or agrammatism (Kertesz et al., 2005; Josephs et al., 2006a,b; Murray et al., 2007; Tree and Kay, 2008; Lee et al., 2011). Limb apraxia and language disorders represent relevant difficulty in daily living in CBS individuals. In spite of language deficits and movement impairment, CBS patients did not usually receive any cognitive or motor rehabilitation treatment.

In the recent years, several studies have reported enhanced cognitive performance in patients with neurological disease after non-invasive brain stimulation (Cotelli et al., 2012b; Flöel, 2014; Civardi et al., 2015).

A promising brain stimulation technique for helping individuals with cognitive impairment is transcranial direct current stimulation (tDCS). tDCS delivers a weak polarizing electrical current to the cortex through a pair of electrodes and brain excitability can be increased via anodal stimulation or decreased via cathodal stimulation depending of the polarity of the current flow (Priori, 2003; Nitsche et al., 2008; Nitsche and Paulus, 2011; Dayan et al., 2013). Recently, tDCS has demonstrated to facilitate naming in young subjects (Sparing et al., 2008; Fertonani et al., 2010, 2014; Wirth et al., 2011) and in older subjects (Holland et al., 2011; Fertonani et al., 2014). Moreover, persistent beneficial effects of tDCS have been observed in neurodegenerative and stroke patients (Baker et al., 2010; Cotelli et al., 2011, 2014b; Boggio et al., 2012; Marangolo et al., 2013a,b; Wang et al., 2013; Manenti et al., 2015).

In this study, we investigated whether modulating the activity of the parietal cortex (PARC) can improve naming performance in patients with CBS. We targeted the PARCs because it has been well established that CBS is characterized by pattern of brain atrophy that involves dramatically parietal areas (Armstrong et al., 2013). Moreover, based on neuroimaging evidence, we assumed that the parietal lobe is specifically involved in action naming and motor representations (Perani et al., 1999; Cappa and Perani, 2003; Saccuman et al., 2006; Liljeström et al., 2008; Péran et al., 2010).

Thus, we predicted a selective shortening of action naming reaction times during anodal stimulation applied over the PARC in CBS patients.

## Materials and Methods

### Subjects

Seventeen patients with a diagnosis of possible CBS (Armstrong et al., 2013) were recruited from the Center for Aging Brain and Neurodegenerative Disorders at the University of Brescia and from the Center for Rehabilitation at the Trescore Hospital, Bergamo, Italy.

Each patient underwent an extensive neurological and neuropsychological evaluation, a routine laboratory examination and conventional brain MRI prior to entering the study to exclude potential alternative diagnoses.

For each patient, motor impairment was evaluated by means of the motor section of the Unified Parkinson Disease Rating Scale (UPDRS-III). Instrumental and basic activities of daily living (IADLs and BADLs, respectively) were assessed as well. Possible scores of ADL range from 0 (no impairment) to 6 (total dependence), whereas the possible scores of IADL range from 0 (no impairment) to 8 (total dependence) (Lawton and Brody, 1969).

The work was conducted in accordance with local clinical research regulations and conformed to the Helsinki Declaration. The study was approved by the local ethics committee and informed consent was obtained from all participants prior to the beginning of the experiment.

### Exclusion Criteria

Stringent exclusion criteria were applied as follows: (a) cerebrovascular disorders, previous stroke, hydrocephalus, and intra-cranial mass as documented by MRI; (b) a history of traumatic brain injury or another neurological disease; (c) significant medical problems; (d) confounding psychiatric disorders; (e) clinically known hearing or vision impairment or a past history of alcohol abuse; (f) implanted metal objects; and (g) history of seizures or any contraindication for tDCS (Nitsche et al., 2003).

### Standardized Neuropsychological Assessment

Two trained neuropsychologists, who were blinded to patient experimental conditions, administered the neuropsychological testing, divided into two sessions. Global cognitive impairment was assessed by Mini-Mental State Examination (MMSE). The battery included measures used to assess memory (Story Recall, Rey-Osterrieth Complex Figure Recall, Digit Span), non-verbal reasoning (Raven’s Colored Progressive Matrices), verbal fluency (phonemic and semantic), language comprehension (Token Test), visuo-spatial capacity (Rey-Osterrieth Complex Figure, Copy), praxis abilities (De Renzi ideomotor apraxia Test), attention (Trail Making Test A and B). All of the tests were administered and scored according to standard procedures (Lezak et al., 2004). The results of the cognitive assessments are presented in Table 1.

**Table 1:** **Demographic characteristics and neuropsychological assessment of patients with Corticobasal Syndrome (*N* = 17)**.

Demographic and clinical features
Age (years)		68.9 ± 6.4
Gender (male/female)		9/8
Education (years)		6.4 ± 2.9
Duration of disease (years)		3.8 ± 2.8
Unified Parkinson Disease Rating Scale (UPDRS—III)		21.6 ± 10.2
BADL (unspared functions)		1.0 ± 1.6
IADL (unspared functions)		1.9 ± 2.1
**Neuropsychological Assessment**	**Raw score**	**Adjusted score**	*Cut-off
**Screening for dementia**
Mini mental state examination (MMSE)	26.4 ± 2.7	25.4 ± 2.8	≥24
**Praxia**
Rey-Osterrieth Complex Figure-Copy	**21.6 ± 7.5**	**23.4 ± 7.3**	>28.87
De Renzi test, right upper limb	**50.9 ± 18.9**		>62
De Renzi test, left upper limb	**54.5 ± 15.2**		>62
**Memory**
Rey-Osterrieth Complex Figure-Recall	9.1 ± 4.1	12.4 ± 5.6	>9.46
Story Recall	10.8 ± 4.8	14.0 ± 4.3	>7.5
Digit Span	4.9 ± 0.9	5.3 ± 0.8	>3.5
**Non-verbal reasoning**
Raven-Colored Progressive Matrices	20.0 ± 4.8	25.6 ± 4.8	>17.5
**Attention**
Trail Making Test, A	104.1 ± 74.0	81.3 ± 73.3	<94
Trail Making Test, B	214.8 ± 46.2	135.1 ± 44.6	<283
**Language**
Fluency-Phonemic	22.4 ± 9.0	29.1 ± 8.2	>16
Fluency-Semantic	30.3 ± 9.2	37.1 ± 9.3	>24
Token Test	29.6 ± 3.0	29.7 ± 2.6	>26.25
International Picture Naming Test, correctness
Actions (%)	61.4 ± 17.6
Objects (%)	86.0 ± 10.2

*Cut-off scores according to Italian normative data are reported. Values are mean ± SD. Bold data indicate scores below cut-off.

## Study Design

**Figure 1:**
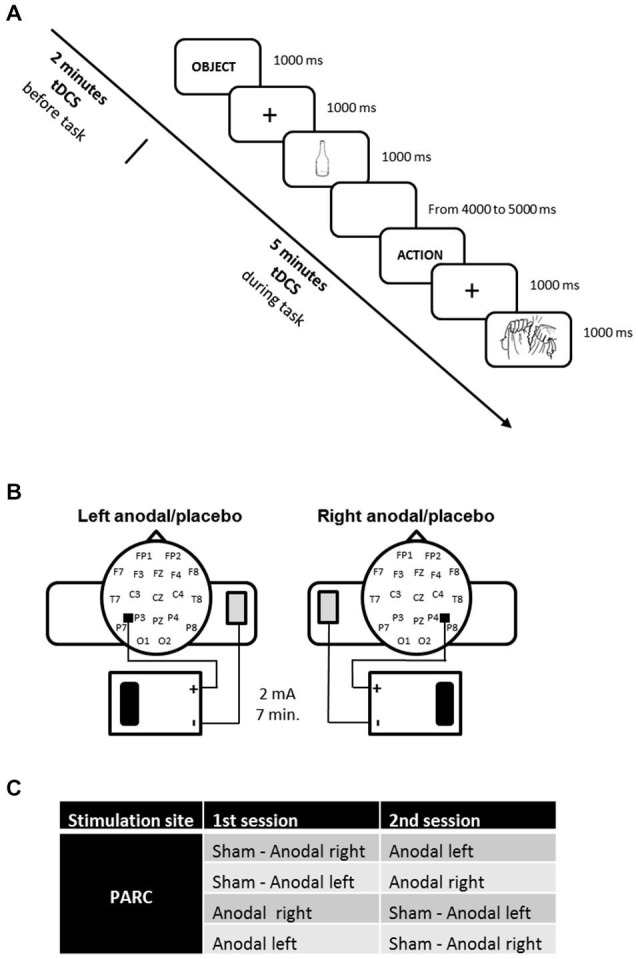
**(A)** Experimental design. The action and object naming task was administered during transcranial direct current stimulation (tDCS), starting 2 min after tDCS beginning. **(B)** tDCS montage on parietal cortices. **(C)** Experimental conditions.

Each patient was subjected to three types of stimulation according to randomization: anodal tDCS over the right PARC, anodal tDCS over the left PARC, and placebo tDCS (see Figure 1A).

The study was a randomized experiment. The patients and the neuropsychologist who assessed patient’s naming performance were blind: they did not know which stimulation patients received (real vs. placebo).

### Experimental Naming Task

During tDCS an action and object naming task has been requested.

#### Stimuli

The stimuli used in the action and object picture naming tasks were taken from the Center for Research in Language-International Picture Naming Project corpus CRL-IPNP (Bates et al., 2000). These items have been tested and normalized in healthy and patient populations across seven different international sites and languages.

We used 108 items (54 actions and 54 objects) as in a previous study using Transcranial Magnetic Stimulation (TMS) in agrammatic Variant FTD (Cotelli et al., 2012a). None of the action stimuli included in the task were associated with the objects selected. The items were divided into three blocks (18 actions and 18 objects each) that were designed for the three stimulation conditions (left PARC, right PARC and placebo stimulation). The frequencies and lengths of the target words, the visual complexity and imageability of the pictures were counterbalanced in the experimental blocks. Ten additional objects and actions were used for a practice block (5 actions and 5 objects).

#### Procedure

Subjects sat in front of a 17-inch monitor that was controlled by a personal computer running Presentation software.^1^ After a frame that indicated the category of the stimulus to the subject (“ACTION” or “OBJECT”), a warning sound 50 ms in duration was presented at the onset of a centrally located fixation cross that was present for 1000 ms. After the disappearance of the fixation cross, the stimulus was presented and remained on the screen for 1000 ms. A blank screen followed for a time varying from 4000 to 5000 ms. The subject’s task was to name, as fast as possible, the stimuli that appeared on the computer screen. Vocal responses were recorded and digitized at 44.1 kHz using the program GoldWave (V. 5.68).^2^ The responses were then analyzed off-line for accuracy (number of correct responses) and vocal reaction times (vRTs). For each stimulus, we calculated the mean response accuracy percentage and the mean vRTs.

### tDCS Procedure

The stimulation was delivered by a battery-driven, constant current stimulator (HDCstim, Newronika, Milan, Italy) through a pair of saline-soaked sponge electrodes (anode electrode: 5 cm× 5 cm; cathode electrode: 6 cm × 8 cm). A constant current of 2 mA was applied for 7 min, starting 2 min before the beginning of the naming task and lasting for the entire task. The current density under the active electrode (0.08 mA/cm^2^) was maintained below safety limits (Poreisz et al., 2007). The electrodes were secured using elastic bands, and to reduce contact impedance, an electroconductive gel was applied under the electrodes before the montage. The anode was placed 5 cm posteriorly and 8 cm laterally with respect to the scalp vertex (at about halfway between P4-P8 and P3-P7) according to the 10–20 EEG international system for electrode placement. The cathode was fixed on the contralateral arm. In the placebo stimulation, the tDCS montage was the same, but the current was turned off 5 s after the start of the stimulation. Therefore, subjects felt the itching sensations below the electrodes at the beginning of the stimulation, making this condition indistinguishable from the experimental stimulation.

The three stimuli blocks corresponded to three stimulation conditions: anodal left, anodal right and sham (i.e., placebo).

The active stimulations (i.e., anodal left and anodal right) were executed on two different days to minimize the likelihood of interference effects (see Figure 1B).

#### Statistical Analyses

Statistical Analyses were performed using Statistica software (version 10; www.statsoft.com). Statistical significance refers to a *p* value of 0.05.

Considering the violation of Normality assumption of the data, we adopted logarithmic transformation of vRTs data and we analyzed log-transformed vRTs.

A 3 × 2 repeated measures ANOVA was used to analyze the mean log-transformed vRTs with two within-group factors: stimulation (placebo, left PARC tDCS and right PARC tDCS) and stimuli (objects and actions). *Post hoc* analysis was carried out by Fisher’s Least Significant Difference (LSD) tests for evaluating pair-wise comparisons among levels of ANOVA significant factors in order to discover which of the comparisons were responsible for rejections in ANOVA test (Hayter, 1986).

Moreover, we analyzed accuracy using two non-parametric Friedman ANOVAs (one for each kind of stimuli, actions and objects).

## Results

### Neuropsychological Assessment

As shown in Table 1, CBS patients exhibited ideomotor apraxia, evaluated with the De Renzi ideomotor apraxia test (De Renzi et al., 1980) and constructional apraxia, evaluated with Rey-Osterrieth Complex Figure-Copy (Caffarra et al., 2002). Otherwise, patients performed within the normal range on memory, non-verbal reasoning, attentional and executive functions. In language domain the patients obtained normal performance in language comprehension and verbal fluency tests. Interestingly, CBS patients obtained lower percentage of correct responses in action naming than in object naming task (*t*_(16)_ = 8.87, *p* < 0.0001).

### tDCS Results

The effects of tDCS over the PARC on object and action naming have been analyzed.

### Reaction Times

**Figure 2:**
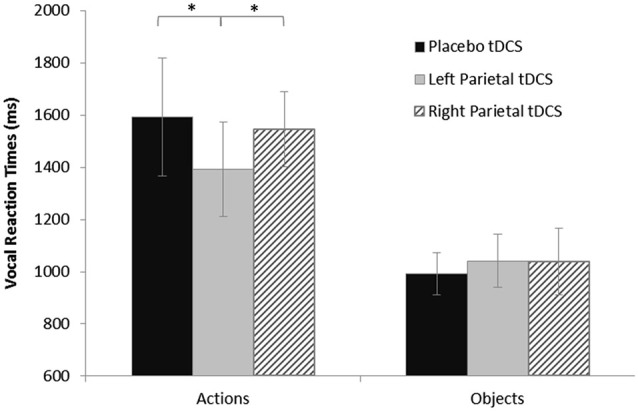
**Vocal reaction times (vRTs) for naming task during each stimulation condition, plotted separately for action and object stimuli**. Asterisks indicate significant effects (*p* < 0.05). vRTs for actions were consistently shorter after left parietal cortex (PARC) than after right PARC or sham/placebo stimulation. No significant differences were observed for object naming. Errors bars indicate mean standard error.

The ANOVA on log-transformed vRTs showed a significant effect of stimulus (*F*_(1,16)_ = 75.73, *p* < 0.0001) and of the interaction between stimulus and stimulation (*F*_(2,32)_ = 4.63, *p* = 0.017). The *post hoc* analysis (LSD) showed that vRTs were significantly higher for actions than for objects (*p* < 0.0001). Furthermore, the *post-hoc* analysis (LSD) revealed a significant shortening after active anodal left PARC stimulation (1392 ± 360 ms) compared to sham (1593 ± 450 ms; left PARC vs. sham, *p* < 0.004) and right (1545 ± 288 ms; left PARC vs. right PARC, *p* < 0.010) stimulation (see Figure 2).

### Accuracy

The analysis of accuracy yielded significant results nor for actions (placebo: 61.4% ± 17.6, left tDCS: 58.9% ± 18.2, right tDCS: 60.8% ± 18.2; *χ*^2^ = 0.54, df = 2, *p* = 0.76) nor for objects (placebo: 85.9% ± 8.1, left tDCS: 87.3% ± 8.7, right tDCS: 88.2% ± 9.4; *χ*^2^ = 2.0, df = 2, *p* = 0.37).

## Discussion

Corticobasal syndrome is a progressive neurodegenerative disease characterized by a specific pattern of brain atrophy in combination with motor and cognitive impairments. Progressive difficulties in language abilities are commonly complaint. These difficulties most frequently involve difficulty with expression of language, such as word finding difficulty (Graham et al., 2003; Grossman et al., 2004; Bak et al., 2005; Cotelli et al., 2006; Kertesz and McMonagle, 2010).

The main purpose of this study was to investigate whether the application of anodal tDCS to the PARC would lead to significant naming facilitation in these patients. To address this question, we compared the effect of anodal tDCS over left and right PARC and placebo tDCS on a picture naming task, observing a significant shortening of vRTs in action naming during left PARC tDCS. Remarkably, the lack of facilitation effects induced by right PARC stimulation provide direct evidence for the specific role of the left PARC in action naming. No effects have been reported for accuracy.

tDCS involves the application of a weak current to the scalp and has the potential to modulate brain networks underlying the performance of a perceptual, cognitive, or motor task (Nitsche et al., 2008). The mechanisms underlying the effects of tDCS are not yet understood but may involve changes in the neuromodulation efficacy of different neurotransmitters (Dayan et al., 2013). The induced excitability changes could persist after the end of the tDCS stimulation, with a duration varying as a function of tDCS parameters (Nitsche and Paulus, 2000). These long-lasting changes are believed to occur at an intracortical level, perhaps mediated through NMDA receptor activity (Liebetanz et al., 2002; Nitsche et al., 2005) and represent a crucial issue for the potential application of this technique into rehabilitation intervention to ameliorate cognitive deficits.

Although lesion studies have indicated a central role of the frontal lobe in verb processing (Cappa and Perani, 2003; Shapiro and Caramazza, 2003), it is now been acknowledged that action processing results impaired also in patients with posterior parietal lesions (Daniele et al., 1994; Silveri and Di Betta, 1997). Neuroimaging studies involving patients have provided evidence for the selective recruitment of different brain areas selectively associated with noun or verb processing (Perani et al., 1999; Shapiro et al., 2006). Specifically, actions apparently evoke stronger activation than objects in the bilateral posterior middle-temporal cortex, in the left temporo-parietal junction and in the left frontal cortex (Liljeström et al., 2008). Moreover, Berlingeri et al. (2008) found bilateral premotor and superior parietal activation during verbs tasks. Nevertheless, recent studies have suggested that the relationship between the grammatical class and the related pattern of brain activation is not clear-cut and must be more thoroughly investigated (Pulvermüller et al., 1999, 2012; Crepaldi et al., 2011; Vigliocco et al., 2011).

A possible explanation of our results is that anodal tDCS effects the brain network involved in action-language and action-representation (Rizzolatti et al., 2001; Cook et al., 2014; Passingham et al., 2014). It is further of interest that the parietal lobes play a crucial role in movement and language, highlighting the likely relationship between action-language and action-representation (Hauk et al., 2004; Tettamanti et al., 2005). Neuroimaging evidence portrait a common fronto-parietal network that underlies action naming and motor representations (Péran et al., 2010). Moreover, several studies have shown that linguistic tasks involving actions activate the same action representation circuits which subserve the execution and the observation of the described actions (Pulvermüller, 2005).

This link between language and action representation has been demonstrated for several linguistic tasks with an involvement of a left-lateralized fronto-parieto-temporal network that closely corresponds to the system for action representation (for example, sentence listening Tettamanti et al., 2005, 2008).

Left PARC has been demonstrated as a crucial area during both observed and imagined grasping (Grafton et al., 1996). Moreover, Passingham et al. (2014) showed that left PARC is crucial in pantomime and suggested that during the evolution of the hominids, the mechanism involved in pantomime could have been used to “name” or request objects.

The direct relationship between language and action has been demonstrated also in Alzheimer’s Disease patients, providing further evidence for a spectrum of concomitant linguistic and praxis deficits in neurological patients (Cotelli et al., 2014a).

Taken together, lesion studies and neuroimaging evidence suggest a strong relationship between naming and motor representation of action. Consistent with previous studies, in the present report CBS patients, characterized by a prevalent parietal atrophy, are more severely impaired in action naming rather than objects (Cotelli et al., 2006). The tDCS effect selectively observed in action naming might be due to the role of PARC for actions or to the baseline high proficiency in object naming.

Several limitations to this pilot study need to be acknowledged. Mainly, the small number of CBS patients, design limitations and the lack of the assessment of long term effects are crucial issues to be addressed. The present preliminary results highlight the improvements induced by a single session of tDCS. Further studies are needed in order to conclusively demonstrate the therapeutic potential of the induction of long-term neuromodulatory effects using brain stimulation.The use of repeated tDCS sessions could be used to investigate the long-term effects of the stimulation, which are particularly interesting in neurodegenerative patients (Flöel, 2014).

These studies, based on larger patient samples and including placebo and randomized control conditions, should be conducted in order to identify the optimal parameters for a useful combined (language training plus tDCS) treatment protocol (Brunoni et al., 2012). Furthermore, other brain stimulation techniques, as TMS, should be tested in CBS (Civardi et al., 2015), comparing the effects of these two stimulation techniques (Fregni and Pascual-Leone, 2007). Finally, a further limitation of the present study is represented by the lack of an effect on accuracy: further studies, including patients at different stages of disease, should investigate this issue.

tDCS technique could be further tested as an effective treatment strategy for language disturbances in CBS patients since our data support the potential usefulness of brain stimulation as a tool for the promotion of neuroplasticity and the development of novel neurorehabilitation strategies.

## Conflict of Interest Statement

The authors declare that the research was conducted in the absence of any commercial or financial relationships that could be construed as a potential conflict of interest.

## Footnotes

^1^www.neurobs.com

^2^www.goldwave.com
